# A Truncated Nef Peptide from SIVcpz Inhibits the Production of HIV-1 Infectious Progeny

**DOI:** 10.3390/v8070189

**Published:** 2016-07-07

**Authors:** Marcela Sabino Cunha, Thatiane Lima Sampaio, B. Matija Peterlin, Luciana Jesus da Costa

**Affiliations:** 1 Departamento de Virologia—Instituto de Microbiologia, Universidade Federal do Rio de Janeiro, Av. Carlos Chagas Filho 373—CCS—Bloco I, Rio de Janeiro 21941-902, Brazil; marcela.scw@gmail.com (M.S.C.); ljcosta@micro.ufrj.br (T.L.S.); 2 Departments of Medicine, Microbiology and Immunology, University of California, San Francisco, 533 Parnassus Avenue, San Francisco, CA 94143, USA; Matija.Peterlin@ucsf.edu

**Keywords:** SIVcpz, HIV, Nef, truncated Nef peptide

## Abstract

Nef proteins from all primate Lentiviruses, including the simian immunodeficiency virus of chimpanzees (SIVcpz), increase viral progeny infectivity. However, the function of Nef involved with the increase in viral infectivity is still not completely understood. Nonetheless, until now, studies investigating the functions of Nef from SIVcpz have been conducted in the context of the HIV-1 proviruses. In an attempt to investigate the role played by Nef during the replication cycle of an SIVcpz, a Nef-defective derivative was obtained from the SIVcpzWTGab2 clone by introducing a frame shift mutation at a unique restriction site within the *nef* sequence. This *nef*-deleted clone expresses an N-terminal 74-amino acid truncated peptide of Nef and was named SIVcpz-tNef. We found that the SIVcpz-tNef does not behave as a classic *nef*-deleted HIV-1 or simian immunodeficiency virus of macaques SIVmac. Markedly, SIVcpz-tNef progeny from both Hek-293T and Molt producer cells were completely non-infectious. Moreover, the loss in infectivity of SIVcpz-tNef correlated with the inhibition of Gag and GagPol processing. A marked accumulation of Gag and very low levels of reverse transcriptase were detected in viral lysates. Furthermore, these observations were reproduced once the tNef peptide was expressed in *trans* both in SIVcpzΔNef and HIV-1WT expressing cells, demonstrating that the truncated peptide is a dominant negative for viral processing and infectivity for both SIVcpz and HIV-1. We demonstrated that the truncated Nef peptide binds to GagPol outside the protease region and by doing so probably blocks processing of both GagPol and Gag precursors at a very early stage. This study demonstrates for the first time that naturally-occurring Nef peptides can potently block lentiviral processing and infectivity.

## 1. Introduction

The closest relatives of the human immunodeficiency virus type 1 (HIV-1) are the simian immunodeficiency virus infecting wild-living chimpanzees (SIVcpz) [1,2] and gorillas (Gorilla gorilla gorilla, SIVgor) [3], while the HIV type 2 (HIV-2) originated from cross-species transmissions of sooty mangabeys infecting viruses (SIVsmm) [4,5]. All of these viruses encode the *nef* gene, which is located at the 3′ end of the genome. Nef is a membrane-associated protein of 25–37 kDa, in which the amino-terminal myristoylation is essential for its localization in cellular membranes [6,7] and function [8,9,10].

The interference of Nef with trafficking of cell surface proteins is its most well-characterized ability. Both SIV and HIV Nef trigger the endocytosis of CD4 from the surface of infected cells to either avoid super infection [11,12] or prevent trapping of the budding viral particles [13]. Regardless of its role in CD4 downregulation, HIV-1 Nef has also been shown to enhance virus infectivity and replication in CD4-negative cells [14,15,16]. Indeed, wild-type (WT) HIV-1 and SIVmac are more infectious than mutants lacking Nef expression (Δ*nef*) [13,17,18,19,20]. Thus, infectivity enhancement and CD4 downregulation activities of Nef can be dissociated [15,16,21]. Nevertheless, the mechanism underlying the influence of Nef in viral infectivity is not understood. More recently, it was demonstrated that Nef counteracts a cellular restriction factor named SERINC [22,23], and this function could be related to the increase in viral progeny infectivity. However, other functions of Nef could also contribute to its influence in viral infectivity.

Nef is incorporated into virions and is processed during maturation by the viral protease [24,25,26], although the functional significance of this cleavage is still not known. Nevertheless, Nef might have an important role during the production of HIV-1 and SIV progeny. Consistent with this idea, our group previously demonstrated that Nef interacts with the viral structural polyprotein GagPol of HIV-1 [27] and influences the viral protease activity and the incorporation of the capsid (CA) protein into viral particles [28].

Whereas it has been demonstrated that the SIVcpz-Nef protein rescues the infectivity of a nef-deficient HIV-1 when added in *trans* [29], until now, no data have been available about the role played by SIVcpz-Nef during the replication cycle of SIVcpz. To investigate this, we generated a nef-defective infectious clone that still expresses an N-terminal 74-amino acid truncated peptide of Nef (SIVcpz-tNef). We found that SIVcpz-tNef behaves differently from classic HIVΔNef infectious clones that still express truncated Nef peptides. Markedly, SIVcpz-tNef progeny were completely non-infectious. The loss in infectivity correlated with the inhibition of Gag and GagPol processing. These observations were reproduced once the tNef peptide was expressed in *trans* both in SIVcpzΔNef and HIV-1WT expressing cells, demonstrating that this phenotype is related to the presence of the tNef peptide and that this peptide is a dominant-negative. This study demonstrates that a Nef peptide derived from SIVcpz can potently act against the processing and release of SIVcpz and HIV-1 viral progeny.

## 2. Methods

### 2.1. Cell Lineages

HEK 293T cells were obtained from the American Type Culture Collection (Manassas, VA, USA), and TZM-bl and Molt 4 (clone 8) cells were obtained from the NIH AIDS Reagent Program Division of AIDS [30,31]. Hek-293T and TZM-bl cells were maintained in DMEM with 10% fetal bovine serum, 1% penicillin/streptomycin and Molt 4 cells in RPMI 1640 medium, with 10% fetal bovine serum, 1% penicillin/streptomycin and 1% of GlutaMAX.

### 2.2. Expression Plasmids

**Figure 1:**
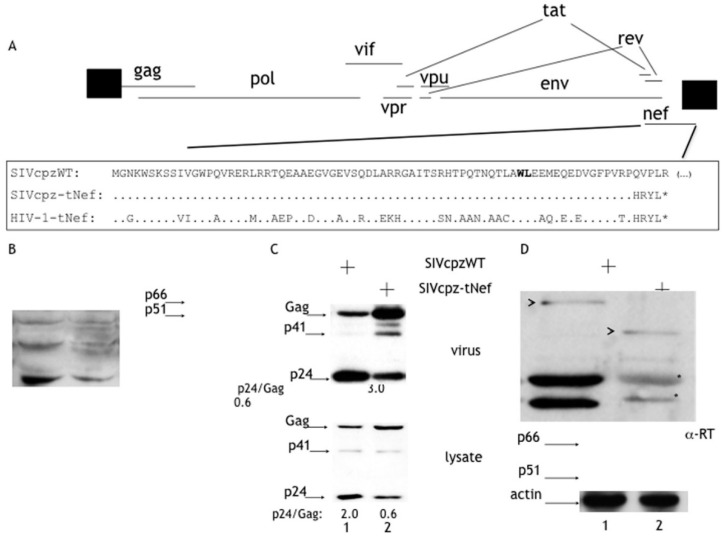
Schematic representation of the SIV chimpanzee (SIVcpz) constructs. (**A**) The genomic organization of SIVcpz and HIV-1 is represented. All open reading frames are shown, and the N-terminal of the Nef ORF is expanded in the box. Sequence coding for the N-terminal domain of SIVcpz and HIV-1 Nef is disrupted to generate the truncated mutants SIVcpz-tNef and HIV-1-tNef. The N-terminal domain of Nef from SIVcpzWT is used as a reference sequence. The SIVcpz-tNef and HIV-1-tNef peptides have a truncated amino acid sequence (HRYL) followed by a premature termination codon. The protease cleavage site within Nef is highlighted (WL), and the arrows indicate stop codons; (**B**) SIVcpz-tNef progeny are non-infectious. Viral infectivity was measured by subjecting the TZM-bl indicator cell line to serial dilutions of normalized supernatants of transfected Hek-293T cultures, and 48 h later, the number of blue foci was scored. Error bars are the standard deviation of the mean. The results are representative of five independently-produced sets of viral stocks; (**C**) Cell-free supernatants from Hek293T cells expressing SIVcpzWT and SIVcpz-tNef were harvested and concentrated through a 20% sucrose cushion. Lysates of Hek293T cells expressing SIVcpzWT and SIVcpz-tNef were harvested 48 h after transfection. Viral and cell lysates were processed for Western blot with polyclonal α-p24, which recognizes Gag (55 kDa), p41-MACA (41 kDa) precursors and the mature p24-CA (24 kDa) protein indicated by arrows. SIVcpz-tNef had a noticeable accumulation of Gag and the partially-processed precursors p41 both in virus and cell lysates. As a measure of the processing efficiency, the p24/Gag ratio was calculated. SIVcpz-tNef processing was reduced by 5-fold in viral lysates and 3-fold in cell lysates; (**D**) The expression of the Pol-associated proteins was also evaluated, viral and cellular lysates were processed for Western blotting with polyclonal α-RT, which recognizes RT (51 kDa and 66 kDa) and unprocessed or partially-processed GagPol. Arrowheads point to partially-processed GagPol of 128 kDa in SIVcpzWT and 110 kDa in SIVcpz-tNef. No RT products were observed in viral and cell lysates of SIVcpz-tNef. Asterisks point to two unspecific bands, and α-actin was used as the loading control.

The replication-competent SIVcpzWT infectious clone derived from the GAB2 virus isolate was obtained from Beatrice Hahn (University of Pennsylvania, Philadelphia, PA, USA). To generate the *nef*-truncated SIVcpz (SIVcpz-tNef), the SIVcpzWT provirus was digested with BSU36I and used for a fill in reaction with Klenow DNA polymerase I (Epicetre Biotechonogies, Madison, WI, USA), followed by T4 DNA ligation (Invitrogen, Carlsbad, CA, USA). Thus, the SIVcpz-tNef provirus carries a two-nucleotide insertion (ca) at nucleotide position 219 of the *nef* gene, generating a truncated peptide encompassing the first 74 amino acids of the Nef protein, followed by 4 extra amino acids (HRYL) derived from the nucleotide insertion and a stop codon. The HIV-1 infectious clones NL4-3 (HIV-1) and their *nef*-deleted cognate NL4-3 ΔNef (HIV-1ΔNef) [32], as well as the NL4-3 vector expressing GFP via IRES (pBR43IeG-nef+) [33] were obtained through the NIH AIDS Reagent Program, Division of AIDS. The NL4-3 vector was used in site-directed mutagenesis reactions to generate HIV-1 nef-truncated (HIV-1-tNef) provirus. HIV-1-tNef harbors an equivalent Nef truncated peptide as the pSIVcpz-tNef. All other plasmid constructions are described in the Supplementary Material Section and are depicted in Figure 1. All of these constructs were used to transiently transfect or electroporate the indicated cell lines either with Lipofectamine 2000 (Invitrogene), FuGENE 6 (Promega, Fitchburg, WI, USA) or using the Neon Transfection System (Life Technologies, Carlsbad, CA, USA). Details about the transfections and electroporation are described in the Supplementary Material Section.

### 2.3. Infectivity Assays

Cell-free supernatants were submitted to ELISA p24, using the Retrotek HIV-1 p24 antigen ELISA Kit (Zeptometrix, Buffalo, NY, USA), performed according to the manufacturer’s instructions. Supernatants were used for the TZM-bl infectivity assay.

### 2.4. Western Blotting

Primary antibodies were: anti-β-actin (Sigma-Aldrich, St. Louis, MO, USA), anti-V5 (Invitrogen), anti-CA(p24) and anti-RT (NIH AIDS Reagent Program). Secondary antibodies were HRP-anti-mouse IgG and HRP-anti-rabbit IgG (GE Healthcare, UK). HRP-linked, goat anti-mouse IgG Dye800 was used (LI-COR, Lincoln, NH, USA). Protein bands were visualized by chemiluminescence (Thermo Scientific, Waltham, MA, USA) followed by X-ray film exposure or by using the LI-COR Odyssey Imaging System and analyzed by using the instrument software. For band quantification, X-ray films were submitted to densitometry, using the macro Gel Plot2 of Scion Image software.

### 2.5. Flow Cytometry

The proportion of co-electroporated Molt 4 cells positive for HIV-1 was measured by the number of GFP+ cells. The cellular viability was evaluated by propidium iodide (PI) (BD Biosciences, Franklin Lakes, NJ, USA). The samples were acquired and analyzed with FACSCalibur^®^, and data were processed using CellQuest software (BD Biosciences).

### 2.6. Proteasome and Lysosomal Inhibition Assay

The day before, 2.2 × 10^6^ HEK-293T cells were seeded in 100-mm dishes. The next day, 6 μg of SIVcpz-WT or SIVcpz-tNef clones were transfected into cells using Lipofectamin 2000 (Invitrogen). Twenty four hour post-transfection, the transfection medium was replaced by fresh medium with 5 μM of the proteasome inhibitor MG132 (Sigma-Aldrich), or 50 nm of bafilomycin A1 (Sigma-Aldrich), or with 1% of DMSO alone. After 5 h, cell-free supernatants and lysates were collected and processed as described above.

### 2.7. Lopinavir Treatment

The day before, 2.2 × 10^6^ HEK-293T cells were seeded in 100-mm dishes. The next day, 6 μg of SIVcpz-WT or SIVcpz-tNef clones were transfected into cells using Lipofectamin 2000 (Invitrogen). Five hours after transfection, the medium was replaced by fresh medium with 28 nM of lopinavir or 1% of DMSO alone. After 24 h, cell-free supernatants and lysates were collected and processed as described above.

## 3. Results

### 3.1. A Nef-Truncated Peptide from SIVcpz Inhibits Gag Processing and Viral Infectivity

Although the Nef protein from SIVcpz also increases viral infectivity [29], the current data are derived from experiments in which the loss of infectivity of a *nef*-deleted HIV-1 provirus is rescued in *trans* by the SIVcpz-Nef protein. To further evaluate the role of the SIVcpz-Nef protein in the replication of an SIVcpz infectious clone, a frame shift truncation within the *nef* gene (SIVcpz-tNef) was introduced within a unique restriction site at position 219 of the *nef* gene, generating a truncated peptide encompassing the first 74 amino acids of the Nef protein followed by four extra amino acids (HRYL) (Figure 1A). The infectivity of SIVcpzWT and SIVcpz-tNef cell-free supernatants from Hek-293T producer cells was evaluated in the TZM-bl indicator cell line (Figure 1B). Blue foci were promptly identified in TZM-bl cells upon infection with SIVcpzWT, making this cell line suitable for titration of SIVcpz viruses. However, supernatants of Hek-293T cells expressing SIVcpz-tNef repeatedly produced no infectious viruses (Figure 1B). Analyses of these supernatants revealed that SIVcpz-tNef accumulated Gag and reduced p24 levels when compared to the SIVcpzWT (Figure 1C). For instance, the CA/Gag relationship, which directly measures viral protease processing, had a five-fold reduction in SIVcpz-tNef. Accumulation of Gag was more pronounced in viral particles than in cell lysates (Figure 1C, compare the upper and lower panels; Figures S1 and S2), but it was still noticed in cell lysates of SIVcpz-tNef-expressing cells.

The amounts of Gag and p24 incorporated into viral particles were measured by quantitative fluorescence-based Western blot and found that processing of Gag in SIVcpz-tNef was inhibited by five-fold when compared to the SIVcpzWT counterpart (Figure S1); while in viral particles, it was more pronounced, achieving an average of 40-fold reduction (Figure S1). The fact that SIVcpz-tNef progeny were completely non-infectious was surprising, since, as shown in Figure 1C, significant levels of processed p24 capsid protein were released into the supernatant of Hek-293T cells.

Due to this lack of infectivity and the processing defect observed in SIVcpz-tNef, the content of enzymatic proteins within viral particles and cells lysates was investigated. The presence of the RT heterodimer (p66/p51) could not be observed in viral particles of SIVcpz-tNef, and a spurious precursor of 111 kDa was consistently detected (Figure 1D). Furthermore, RT was poorly detected in cell lysates (Figure 1D). Moreover, cell lysates were analyzed for the presence of GagPol to determine whether low levels of RT in cells are due to the non-expression or inhibition of processing of the GagPol precursor. We could observe that, albeit in a lower level when compared to SIVcpzWT, GagPol is expressed in SIVcpz-tNef. Interestingly, spurious GagPol processing intermediates were indeed detected in SIVcpz-tNef viral particles by using the anti-CA antibody (Figure S2). The data collectively pointed out to a defect in Gag and GagPol processing for the SIVcpz-tNef provirus, which explains the presence of non-infectious viral particles.

**Figure 2:**
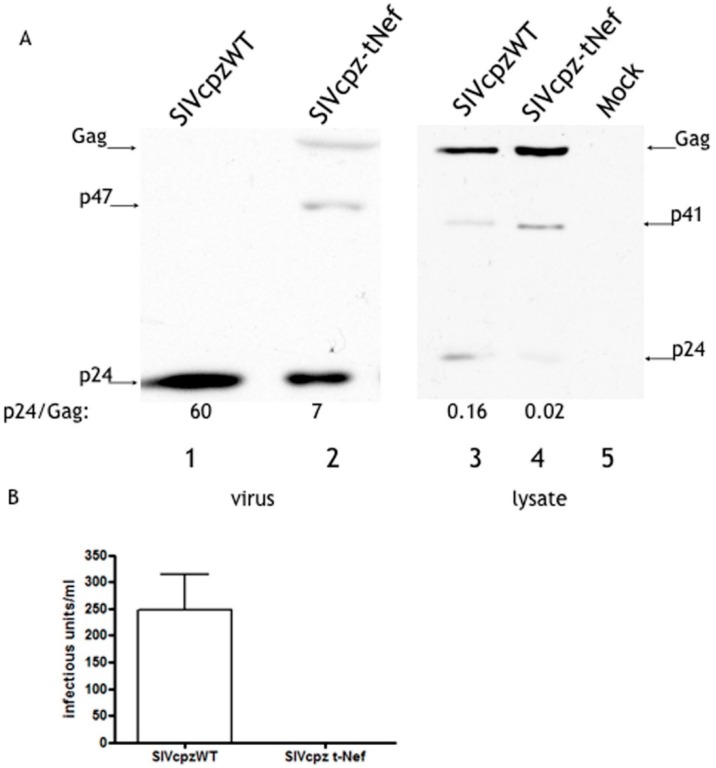
The SIVcpz-tNef phenotype is reproducible in Molt cells. (**A**) Concentrated cell-free of Molt cells transiently transfected with SIVcpzWT and SIVcpz-tNef were processed for Western blot with polyclonal α-CA; (**B**) the infectivity of normalized viral stocks was measured by the TZM-bl assay. Error bars are the standard deviation of the mean. The results are representative of four independent experiments.

SIVcpz-tNef expression in Molt cells led also to the accumulation of Gag and lesser amounts of CA proteins in viral particles when compared to SIVcpzWT (Figure 2A), again leading to the production of non-infectious viral progeny (Figure 2B). In conclusion, the SIVcpz-tNef phenotype previously observed is not cell-type dependent and can also be observed in a lymphocyte T CD4+ cell line.

### 3.2. The Loss of Infectivity of SIVcpz is Due to the tNef Peptide

The infectivity defect in *nef*-deleted HIV-1 isolates is usually reverted by complementing Nef in *trans* in the producer cells [14,34,35]. To revert the tNef phenotype, we added the SIVcpz-Nef protein in *trans* and assayed for viral particle production and infectivity. The defects in Gag processing, the absence of RT sub-unities, and viral infectivity were not rescued by adding increasing amounts of SIVcpz-Nef to the SIVcpz-tNef producer cells (Figure S3).

**Figure 3:**
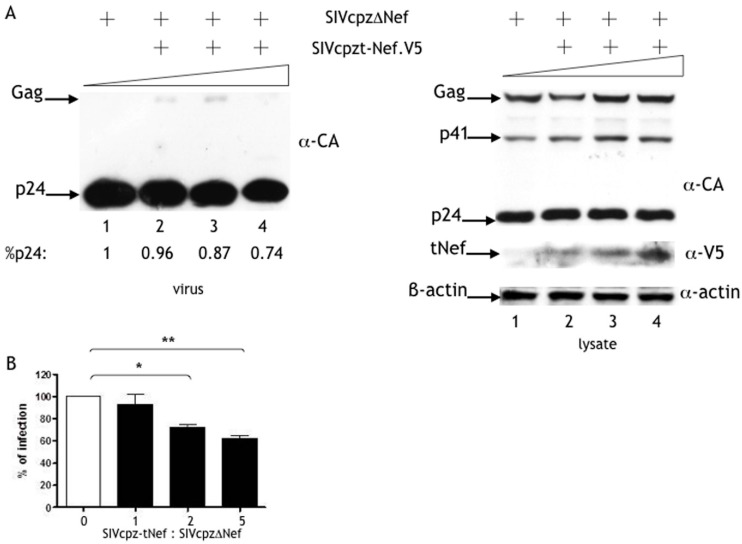
The processing defect and the loss of infectivity of SIVcpz-tNef is due to the tNef peptide. Hek-293T cells were co-transfected with SIVcpzΔNef provirus DNA and the pcDNA.tNef.V5 vector at different ratios (1:0, 1:1, 1:2 and 1:5). Differences in the amount of plasmid DNA in each transfection were compensated by the addition of an empty vector. (**A**) Western blot of concentrated viral particles and cell lysates using polyclonal α-CA, monoclonal α-V5 for the expression of the tNef peptide conjugated to V5 epitope in cell lysates, and α-β-actin as the loading control. Lanes 1–4 are representative of the 1:0, 1:1, 1:2 and 1:5 ratios of SIVcpzΔNef to pcDNA.tNef.V5 vectors, respectively; (**B**) supernatants were used in infectivity assays with the TZM-bl and the number of infected cells was counted and represented as the percentage of the SIVcpzΔNef control (* *p* = 0.011; ** *p* = 0.005). Results represent three independent experiments.

In order to confirm that the tNef peptide was responsible for the SIVcpz-tNef phenotype, a V5-tagged SIVcpz-tNef (tNef.V5) expression vector was generated and co-expressed with the SIVcpzΔNef molecular clone. A decrease in the levels of released p24 was noticed in cell-free viral particles with the increased amounts of SIVcpz-tNef peptide expression (Figure 3A). However, a slightly accumulation of Gag upon tNef.V5 expression was noticed in cell-free viral lysates only at a 1:1 and 1:2 proportion of SIVcpzΔNef to tNef.V5 used. Consistently, at the 1:5 proportion, we observed a more pronounced decrease in p24 release (Figure 3A, left panel). No clear accumulation of Gag upon tNef expression was noticed specifically in cell lysates. Increasing amounts of the SIVcpz-tNef.V5 peptide were readily detected in cell lysates (Figure 3A, right panel). Notably, infectivity was reduced in a dose-dependent way to up to 40% (Figure 3B).

In order to confirm the effect of the SIVcpz-tNef peptide on SIVcpz infectivity, a version of the SIVcpz-tNef peptide harboring a Flag-tag (tNef.Flag) was also tested. Although we were not able to observe the accumulation of Gag in cell-free viral lysates, a pronounced defect in virus release was promptly noticed (Figure S4A). As a control, SIVcpzΔNef was complemented in *trans* with the full-length SIVcpz-Nef. Opposite to what was observed with the tNef.Flag peptide, increasing concentrations of the SIVcpz-Nef did not affect viral release (Figure S4B). Although the effects of tNef.Flag peptide on SIVcpzΔNef processing and infectivity were more discreet than what was observed for tNef.V5 peptide, its effect on reducing viral infectivity was more pronounced.

In fact, while increasing concentrations of tNef.Flag peptide reduced SIVcpzΔNef infectivity up to 80%, infectivity was increased up to three-fold with the addition of increasing concentrations of full-length SIVcpz-Nef.

From these data, we can conclude that the expression of the SIVcpz-tNef peptide in *trans* recapitulates the SIVcpz-tNef phenotype.

### 3.3. The SIVcpz-Nef Protein and the SIVcpz Truncated Peptide Bind to GagPol Polyprotein

**Figure 4:**
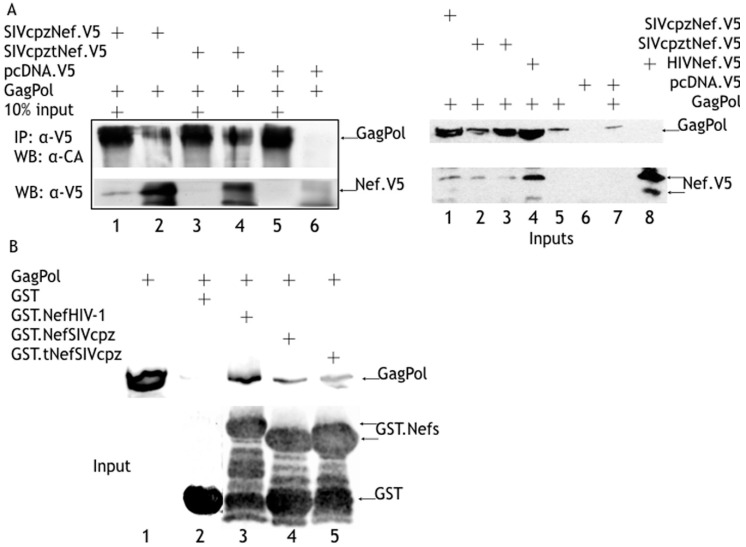
The SIVcpz-Nef protein and the tNef truncated peptide bind to the HIV-1 GagPol polyprotein precursor. (**A**) Hek-293T co-expressing the V5-tagged SIVcpz-Nef protein or the V5-tagged SIVcpz-tNef truncated peptide and a protease-mutated HIV-1 GagPol polyprotein were collected 24 h after transfection; lysates were prepared in RIPA buffer, clarified by low speed centrifugation and immunoprecipitated with anti-V5 antibody followed by protein A/G. Precipitates were washed several times with high salt RIPA buffer. Western blotting of the immunoprecipitates revealed that GagPol was recovered when either SIVcpz-Nef protein or SIVcpz-tNef truncated peptide were present, but not when a pcDNA.V5 empty vector was used; (**B**) GST.SIVcpz-Nef and GST.SIVcpz-tNef fusion protein were purified by glutathione S-transferase columns and incubated with lysates of Hek-293T expressing protease-mutated HIV-1 GagPol polyprotein. Eluates were run in SDS-PAGE, and GagPol was detected by WB with the α-RT antibody. The direct binding of SIVcpz-Nef protein and SIVcpz-tNef truncated peptide to GagPol was confirmed.

Since we have already demonstrated that the HIV-1 Nef protein binds to the GagPol polyprotein precursor [27,36], we hypothesized that the inhibitory effect of the SIVcpz-tNef peptide on Gag and GagPol processing could be due to its retained ability to bind to GagPol. Lysates of Hek-293T co-expressing the V5-tagged SIVcpz-Nef protein or the V5-tagged SIVcpz-tNef truncated peptide and a protease-mutated HIV-1 GagPol precursor were immunoprecipitated with anti-V5 antibody. Western blotting of the immunoprecipitates revealed that GagPol was recovered when either SIVcpz-Nef protein or SIVcpz-tNef truncated peptide were present, but not when a pcDNA.V5 empty vector was used (Figure 4A). Binding of either SIVcpz-Nef protein or SIVcpz-tNef truncated peptide to GagPol was confirmed by GST-pulldown assays GST.SIVcpz-Nef, and SIVcpz-tNef pulled down the GagPol polyprotein from lysates of GagPol transfected Hek-293T cells with the same efficiency as the GST.HIV-1 Nef protein used as the control (Figure 4B).

Interestingly, the apparent molecular weight of the SIVcpz-tNef truncated peptides expressed both as V5-tagged in a eukaryotic system (Figure 4A) and as a GST-fusion in a prokaryotic system (Figure 4B) was twice as the expected from its amino acid length. It could be due to the formation of protein dimmers that were inefficiently disrupted with the denaturing conditions of the SDS-PAGE used.

### 3.4. Inhibition of Proteasome or Lysosome Degradation Does Not Rescue GagPol Levels

**Figure 5:**
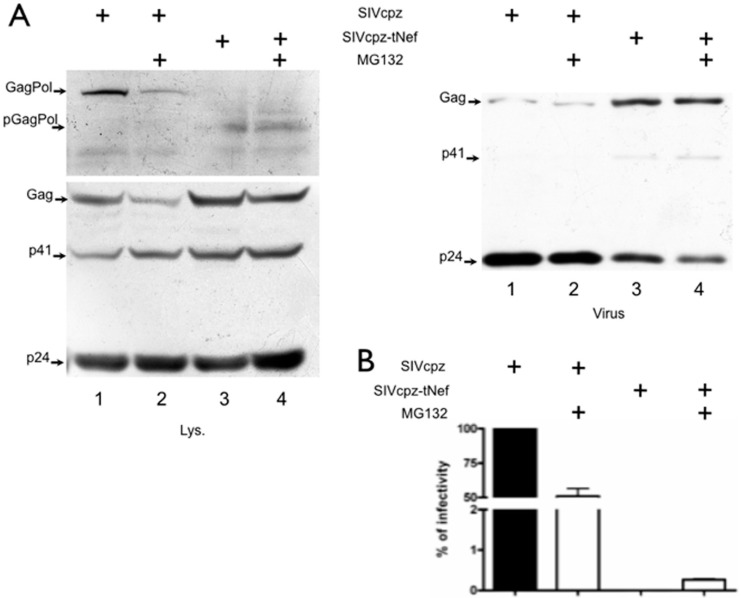
Treatment with proteasome inhibitor does not increase levels of GagPol in SIVcpz-tNef. (**A**) Hek-293T cells were transfected with SIVcpzWT and SIVcpz-tNef clones. After 24 h, the culture medium was replaced, and 5 μM of the proteasome inhibitor MG132 diluted in DMSO or DMSO alone as control were added. After 5 h, supernatants and lysates were collected. Western blotting with polyclonal α-p24 of cell lysates showing full-length and partially-processed GagPol (pGagPol) (top panel); Gag, p41 and p24 (bottom panel). Western blotting of viral lysates with polyclonal α-p24 showing Gag, p41 and p24 (bottom panel); (**B**) Infectivity assays of non-normalized supernatants are represented as the percentage of the non-treated SIVcpzWT control. Results represent three independent experiments.

Since we have observed low levels of GagPol upon SIVcpz-tNef expression and established that the truncated SIVcpz-tNef peptide binds to GagPol, we further investigated the possibility that the truncated tNef peptide would be directing GagPol to the proteasomal degradation. SIVcpzWT and SIVcpz-tNef expressing cells were treated with the proteasome inhibitor MG132. While in lysates of SIVcpzWT expressing cells GagPol was readily detected, no GagPol was present in the lysate of SIVcpz-tNef expressing cells, however, a partially processed GagPol precursor could be observed in this lysate. MG132 treatment partially affected GagPol in SIVcpzWT expressing cells by decreasing its levels. However, GagPol expression from SIVcpz-tNef cell lysates upon MG132 treatment was not rescued (Figure 5A). This result indicates that the low levels of GagPol in SIVcpz-tNef expressing cells are not due to its proteasomal degradation.

It is important to point out that, upon treatment of SIVcpzWT-expressing cells, the release of mature viral particles was reduced by two-fold (Figure 5B). Treatment of SIVcpz-tNef-expressing cells with MG132 also resulted in a decrease in virion release when compared to the SIVcpz-tNef without treatment (Figure 5B). These results demonstrate that the MG132 treatment has an inhibitory effect on SIVcpz release from infected cells.

Moreover, for both viruses, these levels were reduced under MG132 treatment as observed for Gag and p24 (Figure 5B). Since MG132 has a well-document effect on cell growth, the possibility of an overall effect in decreasing protein synthesis and consequently the amount of viruses being released cannot be excluded.

As expected from the results described above for the MG132 treatment, SIVcpzWT infectivity was reduced by 1.8-fold (Figure 5B, consistent with a reduction in the total number of released viruses, indicating that released viruses from MG132-treated cells are as infectious as the viruses released from non-treated cells. Unexpectedly, a minimal recovery of SIVcpz-tNef infectivity upon MG132 treatment was observed, although the infectivity was still very low when compared to the SIVcpzWT (Figure 5C). However, although this could be indicative of the mitigation of the tNef peptide effect, an artifactual effect on infectivity cannot be ruled out.

**Figure 6:**
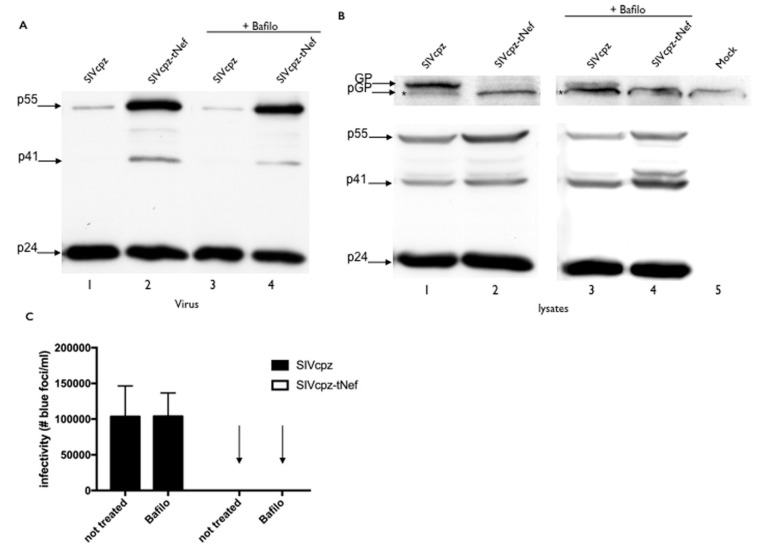
Inhibition of lysosomal degradation does not rescue SIVcpz-tNef GagPol levels and infectivity. (**A**) Hek-293T cells were transfected with SIVcpzWT and SIVcpz-tNef clones. After 24 h, the culture medium was replaced, and 50 nM of bafilomycin diluted in DMSO or DMSO alone as the control was added. After 24 h, supernatants and lysates were collected; (**A**) Western blotting with polyclonal α-p24 of viral lysates showing the accumulation of Gag in SIVcpz-tNef treated and non-treated viruses; (**B**) Cell lysates showing full-length (GP) and partially-processed (pGP) GagPol (top panel); Gag, p41 and p24 (bottom panel); (**C**) Infectivity assays of normalized supernatants. Results are the average of three independent experiments. * Asterisk indicates a non-specific band.

SIVcpzWT and SIVcpz-tNef-expressing cells were also treated with bafilomycin, an inhibitor of lysosomal degradation, and no effect on the increase of GagPol expression or rescuing SIVcpz-tNef Gag processing was observed (Figure 6A,B). Viral infectivity was also unaltered by bafilomycin treatment (Figure 6C). With these data, we can conclude that the reduced levels of GagPol in SVcpz-tNef are not due to either proteasomal or lysosomal degradation directed by the tNef peptide.

From the data described above, we can conclude that the tNef peptide reduces the levels of the GagPol precursor and, by doing that, it blocks processing of the Gag precursor and consequently inhibits virus infectivity. Reduced levels of GagPol are not due to directing either proteasomal or lysosomal degradation directed by the tNef peptide. However, the partial block of virus release upon MG132 treatment alleviated to a certain extent the infectivity defect of SIVcpz-tNef. It could be due to an increase in processing of Gag, an effect that is observed when retroviral release is blocked [37,38]. We hypothesized that, in order to inhibit processing, the tNef peptide would be binding directly to the active site of the viral protease. Therefore, treating SIVcpz-tNef-expressing cells with protease inhibitors would not inhibit processing further. By treating SIVcpzWT and SIVcpz-tNef expressing cells with lopinavir, we observed, for the first time, GagPol in SIVcpz-tNef cell lysates (Figure S5). Therefore, we can conclude that the reduced levels of GagPol are due to either its accelerated processing in the presence of the tNef peptide or a block of processing of GagPol just after a first cleavage, therefore accumulating a partially-processed molecule, as observed previously. In addition, these results indicate that the tNef peptide ought to bind to a different region of GagPol, outside the active site of protease.

### 3.5. The tNef Peptide Is a Dominant Negative

**Figure 7:**
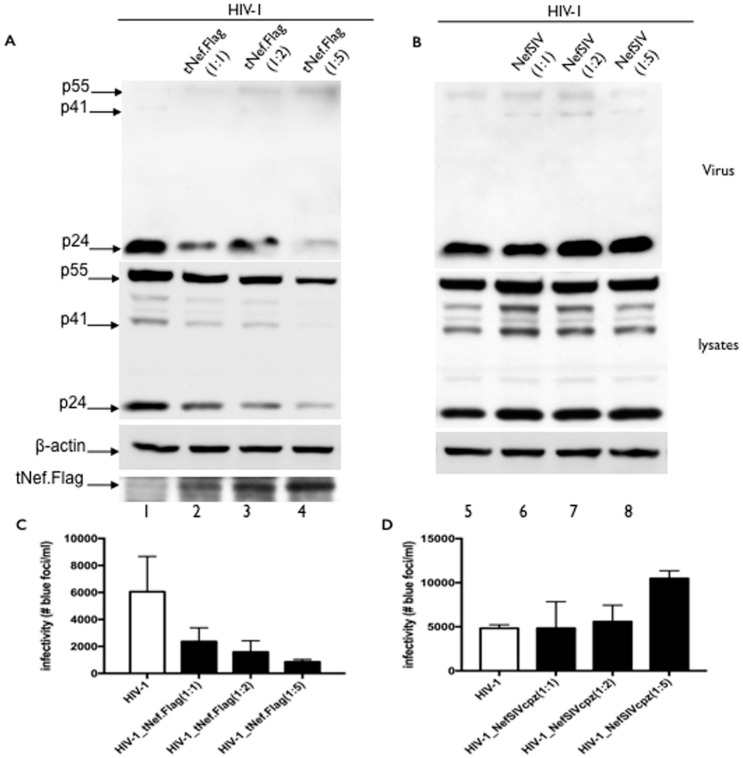
The SIVcpz-tNef peptide is a dominant negative for viral release and infectivity. (**A**) Hek293T cells were co-transfected with 1 μg of HIV-1WT clone and 0, 2 and 5 μg of the SIVcpz-tNef.Flag vector (1:0; 1:2 and 1:5 ratios). Differences in the amount of plasmid DNA in each transfection were compensated by the addition of the empty vector (pCMV.4). Viral and cell lysates were collected 24 h post-transfection. Western blotting of viral particles concentrated by ultracentrifugation in a 20% sucrose cushion, using a α-CA antibody (upper panel). Western blotting of cell lysates using a α-CA, α-actin antibody as the loading control and α-Flag antibody for the expression of tNef.Flag (lower panels); (**B**) As a control, HIV-1 was co-transfected with increasing amounts of the SIVcpz-Nef expression plasmid, as above; (**C**,**D**) Supernatants were used in infectivity assays with the TZM-bl indicator cells, and the number of infected cells was counted and plotted. Numbers bellow each histogram represent the different proportions of the HIV-1WT proviral DNA to the pcDNA.tNef.Flag vector (1:0, 1:2 and 1:5) (**C**) or the SIVcpz-Nef (**D**). Differences in infectivity for the 1:2 and 1:5 co-expression ratio of the SIVcoz tNef.Flag peptide were statistically significant (*p* = 0.0373 for the 1:2 proportion; *p* = 0.028 for the 1:5 proportion).

Dominant-negative HIV-1 Nef F12 protein was previously demonstrated to interfere with the virus production and infectivity of HIV [39,40]. Since defects in Gag/GagPol processing and viral infectivity of SIVcpz-tNef were not rescued in *trans* by the SIVcpz-Nef protein (Figure S3), we hypothesized that this truncated peptide would function as a dominant negative. To test this hypothesis, increasing concentrations of an expression vector for a Flag-tagged or V5-tagged versions of the tNef peptide were added to HIV-1WT expressing cells. Expression of SIVcpz-tNef.Flag peptide at the highest concentration inhibited HIV-1 release and to some extent Gag processing (Figure 7A). By using SIVcpz-Nef as a control, none of these above-mentioned results were observed (Figure 7B); in fact a slight increase in both viral release and Gag processing was noticed upon increasing the expression of the SIVcpzWT Nef protein. HIV-1 infectivity was inhibited in a dose-dependent way with the tNef.Flag peptide (Figure 7C), while the infectivity of HIV-1 was increased upon increasing concentrations of the SIVcpz-Nef protein expression (Figure 7D). We tested the ability of the V5-tagged versions of the tNef peptide to act as a dominant negative during the replication of HIV-1 in the Molt lymphocytic CD4+ cell line. For that, an HIV-1 infectious clone expressing a bicistronic mRNA for Nef and GFP was used; therefore, HIV-1 expressing cells would be positive for GFP. The release (Figure S6A) and infectivity (Figure S6B) of mature HIV-1 progeny was inhibited by six-fold in the presence of the tNef peptide, recapitulating the phenotype observed in 293T cells with the tNef peptide. However, a clear effect on Gag processing could not be observed upon SIVcpz-tNef.V5 co-expression (Figure S6A,C). We consistently observed a 30% reduction in the number of GFP+ cells when the tNef peptide was co-expressed, which was probably due to some cytotoxic effect of this peptide on Molt cells. The same experiments with the SIVcpz-tNef.V5 peptide were performed in Hek-293T cells, and the equal results were observed (Figure S7). The data described above indicate that the SIVcpz-tNef peptide acts as a dominant negative for viral release and infectivity in both non-lymphocytic and TCD4+ cells. We examined whether the dominant-negative effect of the SIVcpz-tNef peptide was also recapitulated upon the co-expression of HIV-1WT and SIVcpz-tNef proviruses. Co-expression of HIV-1WT and SIVcpz-tNef at a 1:1 ratio did not result in any significant reduction in capsid (p24) release and Gag processing (Figure S8A). Nevertheless, a dramatic reduction in viral infectivity was observed (Figure S8B). Moreover, when HIV-1WT and SIVcpz-tNef were co-expressed at a 1:5 ratio, we could observe a defect in Gag processing, CA release (Figure S8A) and a complete inhibition of viral infectivity (Figure S8B). These results confirm that the tNef peptide is a dominant-negative for HIV-1 and SIVcpz replication.

*nef*-deleted mutants of HIV-1 infectious clones have been generated by using the same strategy as the SIVcpz-tNef described herein. For instance, a widely-used *nef*-deleted HIV-1 (NL4-3ΔNef) potentially express a 35-amino acid truncated Nef [32]; however, it does not have a similar phenotype as the SIVcpz-tNef. Given the length difference, we hypothesized that a 74-amino acid tNef version of HIV-1 would have the same effect as the SIVcpz-tNef (Figure 1). Transient expression of the HIV-1-tNef showed the same levels of Gag and processed CA and RT when compared to both HIV-1WT and HIV-1ΔNef (Figure S9). Furthermore, the infectivity of HIV-1-tNef had a 2–3-fold reduction when compared to HIV-1WT, similar to the reduction observed for HIV-1ΔNef (Figure S9). From these data, we can conclude that the inhibitory effect on viral infectivity and Gag/GagPol processing is exclusive for the tNef peptide from SIVcpz. Furthermore, since the HIV-1 tNef peptide also harbors the amino acids HRYL at its C-terminal, we can exclude any contribution of these amino acids to the phenotype of the SIVcpz-tNef.

## 4. Discussion

In the present study, we demonstrated that a truncated peptide from *nef* of SIVcpz can specifically inhibit the processing and, consequently, infectivity of SIVcpz and HIV-1. This peptide targets Gag and GagPol maturation in a dominant negative way. Furthermore, the co-expression of the tNef peptide together with the *nef*-deleted SIVcpz and HIV-1 proviral clones recapitulates the phenotype observed for the SIVcpz-tNef clone, albeit in a lesser extent. It was demonstrated, for the first time, that the SIVcpz-Nef protein has the capacity to bind directly to the HIV-1 GagPol precursor, indicating that the ability of the Nef protein to bind to GagPol is conserved amongst the primate lentiviruses [27,36]. More importantly, results confirmed that the SIVcpz-tNef peptide binds to GagPol both in mammalian cells and in vitro. These results indicate that the SIVcpz-tNef peptide exerts its inhibitory activity directly on to the GagPol precursor polyprotein and consequently affects processing of Gag and viral infectivity in a very efficient way.

The genetic modification, which generated the SIVcpz-tNef clone, had been extensively used to generate most of the *nef*-deleted HIV-1 clones described in the literature, although any effect on viral maturation has never been documented [14,20,41,42]. Truncated Nef-peptides in Nef-deleted HIV-1 are smaller in length; however, the 74-amino acid length truncation in *nef* of HIV-1 generated by us did not reproduce those effects described for the SIVcpz-tNef. Thus, the phenotype of the SIVcpz-tNef peptide, both in Gag/GagPol processing and viral infectivity, is specific. Furthermore, since this HIV-1 tNef peptide also harbors the extra HRYL amino acids at the C-terminus, as the SIVcpz-tNef peptide, the presence of these amino acids does not seem to contribute to the SIVcpz-tNef phenotype. For instance, although detection of either the Flag or the V5-tagged SIVcpz-tNef peptide by Western blotting and immunofluorescence was inefficient, probably due to the instability of the peptide, we were able to verify that its subcellular localization was similar to that of the HIV-1Nef protein. Therefore, the addition of the HRYL stretch of amino acids at the C-terminal of the SIVcpz-tNef peptide did not alter its cellular localization, which is expected for its action as a dominant negative, since it would be present at the same sites as the Nef protein.

It is important to point out that, although the main motifs present within the N-terminal region of Nef, namely the mirystoylation site and the stretch of basic residues involved with membrane targeting of Nef, are conserved between HIV-1 and SIVcpz, the degree of amino acid identity between the SIVcpz-tNef and HIV-1-tNef truncated peptides is only 80.2%. Thus, a low degree of functional similarity is expected. Moreover, the *env* and *nef* ORFs in the SIVcpz genome do not overlap; therefore, any effect of this manipulation on the *env* gene can be excluded.

The notorious phenotype observed for the SIVcpz-tNef proviral clone was the accumulation of Gag and the consequent reduction in the formation of the mature CA protein, especially in cell-free viral particles (Figure 1C). Albeit reduced, some levels of mature p24 can be detected in supernatants of SIVcpz-tNef-expressing cells, which do not correlate with the complete reduction in viral infectivity. This was explained by the fact that RT is absent from viral particles (Figure 1D). Instead, a partially-processed polyprotein precursor of approximately 111 kDa derived from GagPol was consistently detected in cell-free supernatants when we probed with both anti-RT (Figure 1D) and anti-p24 (Figure S2) antibodies, indicating that this precursor harbors both the p24 and RT proteins. The presence of this above-mentioned precursor does not correlate to any partially-processed precursors of GagPol generated during the normal cleavage of this precursor in *cis* or in *trans* [43,44,45] and is consistent with cleavage occurring simultaneously both at the MA/CA and RH/IN. Moreover, the noticed instability of GagPol in tNef-expressing cell lysates was not rescued upon MG132 or bafilomycin treatment; therefore, targeting of GagPol to proteasomal or lysosomal degradation by the SIVcpz-tNef peptide can be ruled out. However, the synergic effect of the protease inhibitor lopinavir on the processing inhibition by the SIVcpz-tNef peptide suggests that this peptide binds to a region of GagPol outside the active site of the protease. Lopinavir treatment made it possible to detect GagPol on cell lysates of SIVcpz-tNef-expressing cells, this observation suggests that, in the presence of the SIVcpz-tNef peptide, GagPol goes through the first cleavage events, which result in the formation of a partially-processed GagPol polyprotein and the consequent disappearance of full-length GagPol. When the protease activity is blocked by a protease inhibitor, these first steps of cleavage do not occur, and GagPol can be detected. The probable mechanism by which SIVcpz-tNef peptide exerts its effects is binding GagPol on the cleavage sites that follow the first events of cleavage or to a region of GagPol that modifies its structure next to these cleavage sites, making them inaccessible and, consequently, blocking a partially-processed GagPol in a determined structure, which will not be processed further. Therefore, the SIVcpz-tNef peptide may be an interesting molecule to target HIV-1 maturation by a different mechanism of the protease inhibitors currently used in the anti-retroviral therapy.

The deleterious effect of the tNef peptide on Gag processing and viral infectivity observed upon co-transfection with SIVcpz and HIV-1 proviral clones did not reproduce the extent of the effect observed for the SIVcpz-tNef provirus. This could be due to a suboptimal proportion of co-transfected cells in transient transfections. Moreover, since different promoters drove the expression of the tNef peptide and Gag and GagPol proteins, different levels of these products could have occurred in co-transfected cells, leading to a less pronounced effect of the truncated peptide. Furthermore, the tags used to detect the SIVcpz-tNef could influence either its stability or function. A more pronounced effect on infectivity, however, was noted when a Flag tag was used. On the other hand, the V5-tagged version of the SIVcpz-tNef peptide had a less pronounced effect and showed some degree of cytotoxicity in Molt cells. In any case, the effect of the tNef peptide in *trans* was dose-dependent and reproducible. Indeed, the co-expression of the SIVcpz-tNef DNA clone with the HIV-1WT infectious clones drastically interfere with viral infectivity, but the visual effects on Gag processing were less pronounced. The same phenomena have been demonstrated upon protease inhibitor treatment of HIV-1 producer cells. Even small effects on the inhibition of Gag processing, observed as low accumulation of Gag in virus-expressing cells, are sufficient to render viral progeny non-infectious. Our results clearly indicate that the phenotype of the SIVcpz-tNef proviral clone is due to the presence of the Nef truncated peptide. However, the data should be confirmed in an optimized cell line stably expressing the tNef peptide in an inducible way.

In the present study, we observed that the inhibition of the ubiquitin-proteasome system impacted on the release of SIVcpzWT progeny. The requirement of free ubiquitin for viral budding has already been demonstrated for HIV, SIV, MLV, RSV and MuLV [37,38,46,47,48]. Although this requirement is not dependent on the site of budding, the late domain context and the ubiquitination of Gag, it seems that the participation of ubiquitin ligases and/or deubiquitinases is crucial for viral release [49,50,51,52,53]. Indeed, the presence of proteasome inhibitors during the post-transcriptional phase of HIV-1 infection affected virion release, leading to net effects on the activity of the viral PR [37]. Nonetheless, the possibility of an overall effect of MG132 in decreasing protein synthesis and consequently the amount of viruses being released cannot be excluded.

The results of this study demonstrate that a peptide derived from the wild-type Nef protein from SIVcpz imposes a dominant negative effect on the processing and infectivity of both SIVcpz and HIV-1 viral progeny. Some examples exist in the literature describing dominant negative HIV proviral clones and/or viral-derived proteins on the maturation and processing steps of the HIV-1 replication cycle [39,54,55,56,57]. These dominant negatives demonstrated severe consequences on the infectivity of the viral progeny. The F12 proviral clone, derived from the NL4-3 laboratory-adapted virus, was demonstrated to be a dominant negative for Gag processing and viral infectivity [39]. This virus harbored mutations at *gag*, *vif* and *nef* genes related to the dominant negative phenotype [54]. Expression of the mutant Nef protein from F12 alone was sufficient to interfere with Gag processing and viral infectivity of other HIV-1 strains in *trans* [39,40]. Dominant negative inhibition of HIV-1 maturation achieved by several mutants of Gag at different PR cleavage sites had highly detrimental effects on the viral infectivity of a wild-type HIV-1 infectious clone [54,55,56,57]. Except for an MA/p24 boundary mutant that completely inhibited the formation of the p24 protein [54,56,57], none of those mutants exerted drastic effects on Gag. Additionally, no effect of these mutants was observed on GagPol processing. The lower impact on Gag processing correlated with the less potent trans-dominant activity of the mutant. However, the mechanism of action of these dominant-negative mutants has not been characterized. Results from these studies allowed the conclusion that the presence of minor amounts of incompletely-processed Gag intermediates affects the post-entry steps of the viral replication cycle. In light of these observations, our data support the idea that very early interference in the proteolytic cleavage of the GagPol precursor by the tNef peptide accounts for the observed phenotype. Therefore, trans-dominant peptides derived from the HIV and SIV genomes could represent potent tools to therapeutically inhibit HIV-1 replication. As demonstrated in this work with the SIVcpz-tNef peptide, targeting a fundamental step of viral replication, already targeted by the protease inhibitors, but by a different mechanism, this molecule could possibly be contributing to increasing the arsenal of molecules to be used in the anti-retroviral therapy.

## 5. Conclusions

In this report we demonstrated that a truncated Nef peptide from SIVcpzGab2 interferes with processing of the Gag polyprotein precursor and abolishes infectivity of SIVcpz viral progeny. The effect of SIVcpz-tNef peptide is recapitulated upon its co-expression with HIV-1, demonstrating that this peptide is a dominant negative for viral infectivity. The SIVcpz-tNef peptide can be explored as an antiviral molecule targeting the maturation step of viral replication and represents a valuable tool to dissect the assembly and maturation steps of HIV-1 and SIVcpz viruses.

## Supplementary Materials

The following are available online at www.mdpi.com/1999-4915/8/7/189/s1, Figure S1: SIVcpz-tNef has a processing defect, Figure S2: Low levels of GagPol are present upon expression of SIVcpz-tNef, Figure S3: SIVcpzNef transcomplementation does not rescue the processing defect of the SIVcpz-tNef molecular clone, Figure S4: A flag-tagged SIVcpz truncated peptide (tNef.Flag) recapitulates the phenotype of the SIVcpz-tNef clone, Figure S5: Treatment of SIVcpz-tNef expressing cells with the protease inhibitor Lopinavir increases levels of GagPol in cell lysates, Figure S6: The SIVcpz truncated SIVcpz tNef peptide inhibits the release of HIV-1 progeny from Molt cells, Figure S7: SIVcpz-tNef.V5 peptide inhibits HIV-1 infectivity, Figure S8: SIVcpz-tNef is a dominant negative for HIV-1WT, Figure S9: The SIVcpz truncated tNef peptide inhibits the release of HIV-1 progeny from Molt cells.

## Abbreviations

AIDS	acquired immunodeficiency syndrome
CA	capsid
Env	envelope
Gag	group-specific antigen
GagPol	Gag-polymerase
HIV	human immunodeficiency virus
Nef	negative factor
SIV	simian immunodeficiency virus
VLP	virus-like particle
MLV	murine leukemia virus
RSV	Rous sarcoma virus
MuLV	Moloney murine leukemia virus
EIAV	equine infectious anemia virus
