# Supplementary material for: A Truncated Nef Peptide from SIVcpz Inhibits the Production of HIV-1 Infectious Progeny
Source: Viruses. 2016 Jul 7;8(7):189. doi: 10.3390/v8070189 (PMC4974524; doi:10.3390/v8070189)
Supplement: Supplementary file 1 [file viruses-08-00189-s001.pdf]

Viruses **2016**, *8*, 189; doi: 10.3390/v8070189

Supplementary Materials: A Truncated Nef Peptide from SIVcpz Inhibits the Production of HIV-1 Infectious Progeny

Marcela Sabino Cunha, Thatiane Lima Sampaio, B. Matija Peterlin and Luciana Jesus da Costa

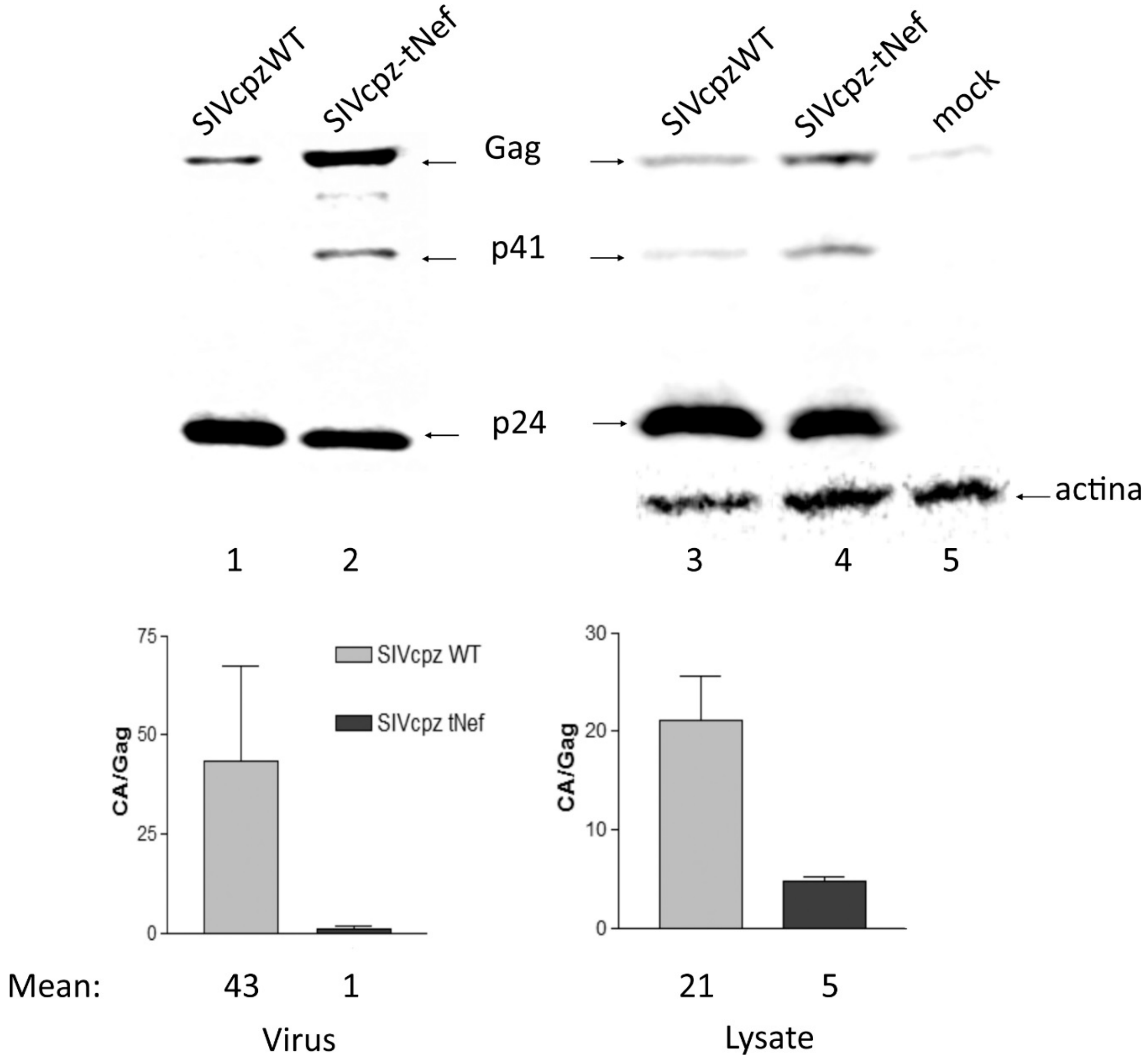

Figure S1. SIVcpz-tNef has a processing defect. HEK 293T cells were transfected with 2 µg of the SIVcpzWT, SIVcpzΔNef and SIVcpz-tNef proviral DNAs. Supernatants and cell lysates were collected 48 h post transfection. Western Blot of viral particles concentrated by ultracentrifugation in a 20% sucrose cushion and cellular lysates using a α-CA antibody. The intensity of each band was measured with the LICOR system. The CA/Gag relationship is represented on the graphics bellow. Primary α-β-actin was used for loading control.

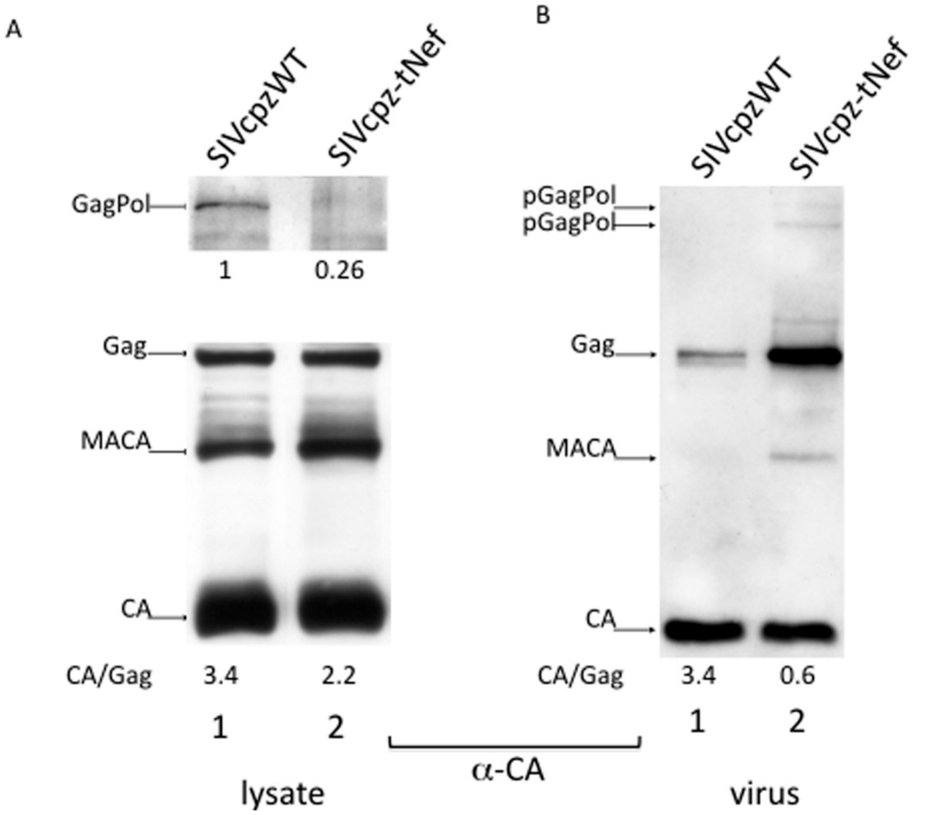

Figure S2. Low levels of GagPol are present upon expression of SIVcpz-tNef. Cell lysates (**A**) and cell-free supernatants (**B**) were used to detect GagPol expression with the polyclonal α-CA, which recognises GagPol, Gag (160 kDa and 55 kDa, respectively) and partially processed GagPol (pGagPol). Values bellow each lane are percentage of GagPol to the control (SIVcpzWT) (**A**, top), and the CA/Gag relationship. This result is representative of 4 experiments.

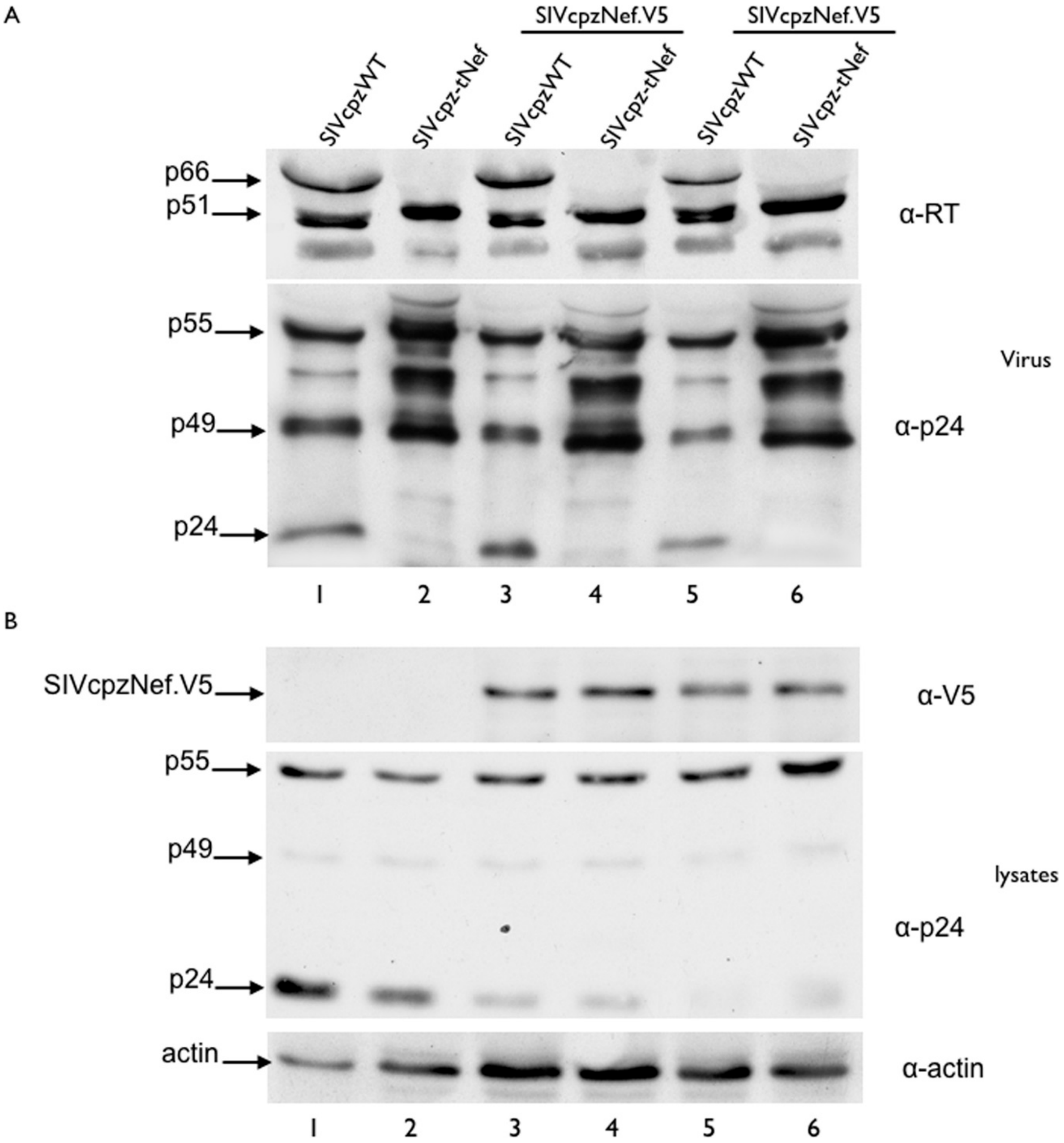

Figure S3. SIVcpzNef transcomplementation does not rescue the processing defect of the SIVcpz-tNef molecular clone. In order to rescue the processing defect and the loss of infectivity of SIVcpz-tNef a expressing vector for SIVcpzNef.V5 was added in trans into SIVcpz-tNef expressing-Hek-293T cells. Co-transfections were performed with two different amounts of the SIVcpz-tNef expressing vector at a 1:1 and 1:5 ratio of the SIVcpz-tNef molecular clone to the Nef expressing vector. The SIVcpzWT molecular clone was used as a control. Differences in the amount of plasmid DNA in each transfection were compensated by the addition of an empty vector (pcDNA3.1) to a final concentration of 3 μg. (**A**) Cell-free supernatants and (**B**) cells lysates were harvested 48 h after transfection. Viral particles were concentrated by ultracentrifugation in a 20% sucrose cushion, viral lysates were processed for WB using an α-CA and α-RT antibodies. Cell lysates were processed for WB using an α-V5 antibody to check for SIVcpzNef expression; α-CA polyclonal antibody and α-actin monoclonal antibody as a load control. RT p66/p51 and Capsid p24 bands were not observed in viral lysates of SIVcpz-tNef even with the highest concentration of SIVcpzNef added; (**C**) Viral stocks were normalized and viral infectivity was assayed by TZM-bl indicator cell assay. The SIVcpz-tNef progeny either not complemented or complemented in *trans* with SIVcpzNef was non-infectious. Results were average of 3 independent experiments. * Asterisks mark a non-specific band.

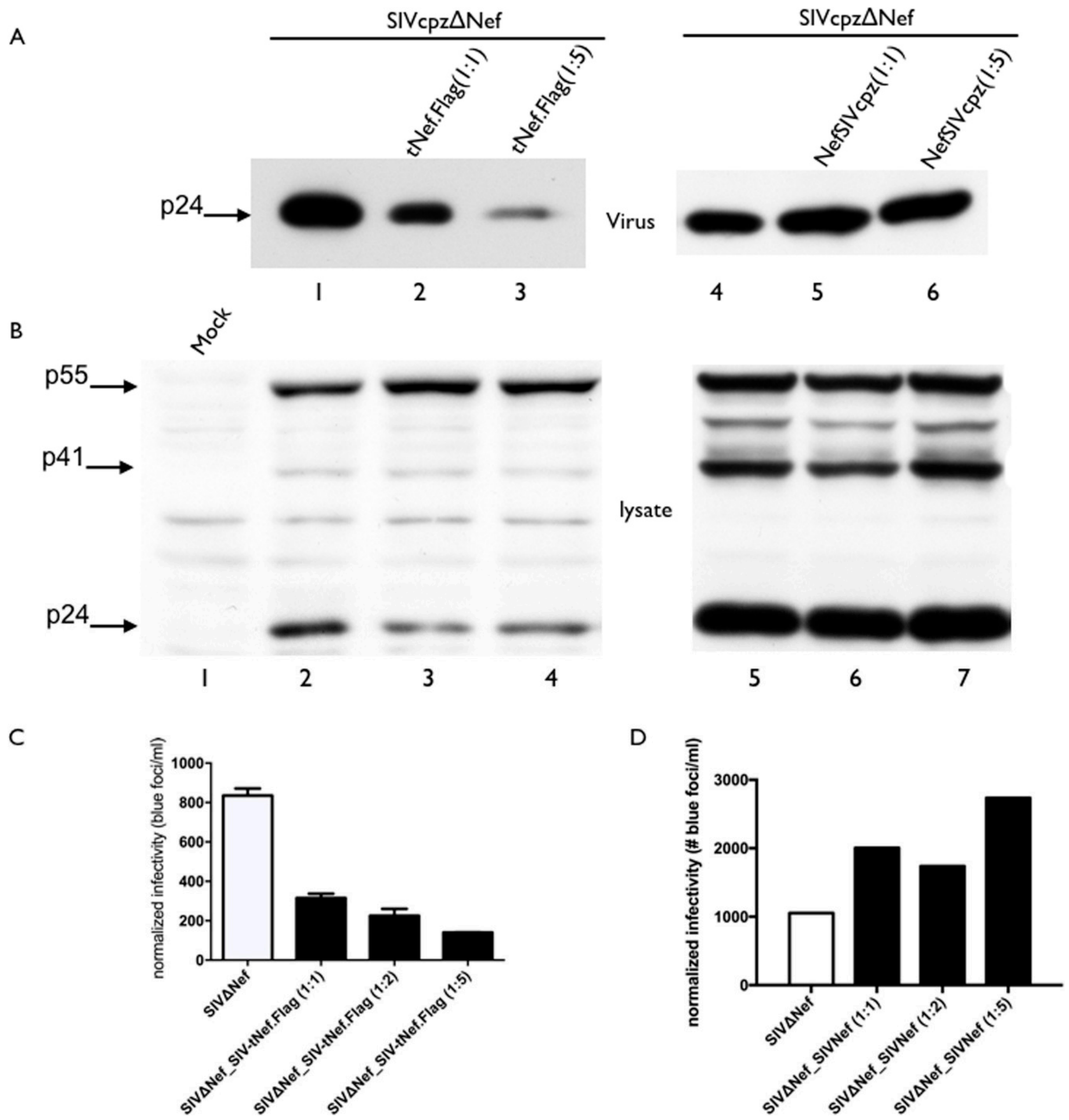

Figure S4. A flag-tagged SIVcpz truncated peptide (tNef.Flag) recapitulates the phenotype of the SIVcpz-tNef clone. In order to recapitulate the effect of the t-Nef peptide during the replication cycle of a SIVcpz molecular clone, Hek-293T cells were co-transfected with different proportions (1:1 and 1:5), of the *nef-deleted* SIVcpz (SIVcpzΔNef) and the expression vector for the tNef.Flag peptide. Differences in the amount of plasmid DNA in each transfection were compensated by the addition of an empty vector (pcDNA3.1) to a final concentration of 3 μg. The SIVcpzΔNef molecular clone was also complemented in *trans* with the SIVcpzNef as a control. (**A**) Cell-free supernatants and (**B**) cells lysates were harvested 48 h after transfection. Viral particles were concentrated by ultracentrifugation in a 20% sucrose cushion, viral and cell lysates were processed for WB using an α-CA antibody. Less mature viruses were released from cells expressing the tNef.Flag peptide in a dose-depended way. The expression of the SIVcpzNef (lanes 1–3), however, slightly increased virus release (lanes 4–6), as observed in (**A**). A discreet increase in p55Gag levels upon expression of increasing leves of tNef.Flag, and a concomitant decrease in processed p24 was noticed in cell lysates (**B**); (**C** and **D**) Viral stocks were normalized and viral infectivity was assayed by TZM-bl indicator cell assay. The values are the average of triplicate assays of each viral stock; error bars are the standard deviation of the mean. Values were statistically significant (p = 0.006 for the 1:1 ratio; p = 0.0033 for the 1:2 ratio; p = 0.0227 for the 1:5 ratio of SIVcpzΔNef to tNef.Flag transfection). The increasing expression of the tNef.Flag peptide reduced SIVcpzΔNef infectivity by 73%–84%, while expression of SIVcpzNef increased viral infectivity up to 3-fold.

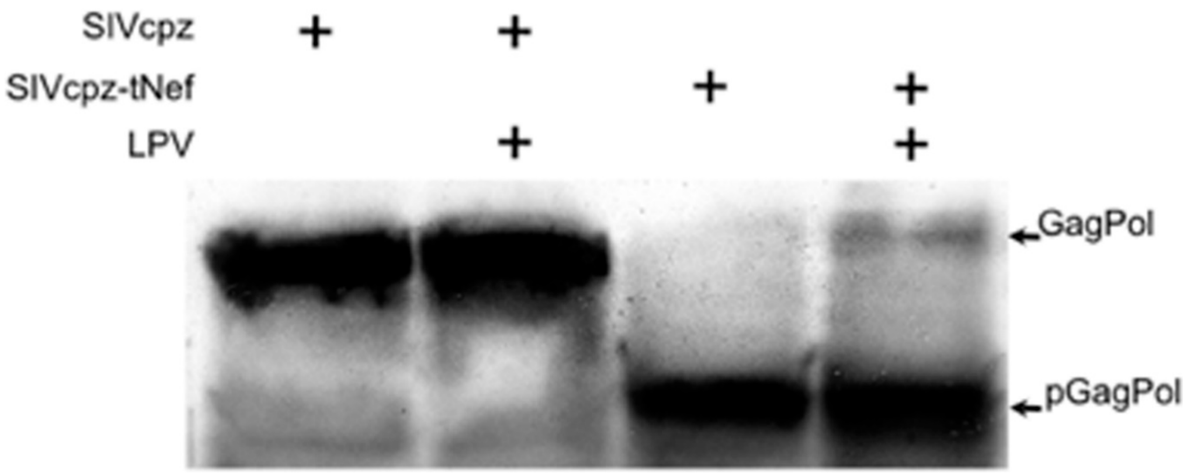

Figure S5. Treatment of SIVcpz-tNef expressing cells with the protease inhibitor Lopinavir increases levels of GagPol in cell lysates. Hek-293T cells were transfected with SIVcpzWT and SIVcpz-tNef clones. After 5 h, the culture medium was replaced and 28 nM of Lopinavir or medium alone as control were added. After 24 h, lysates were collected. Western-Blotting with polyclonal α-p24 of cell lysates showing full-length (GagPol) and partially processed GagPol (pGagPol).

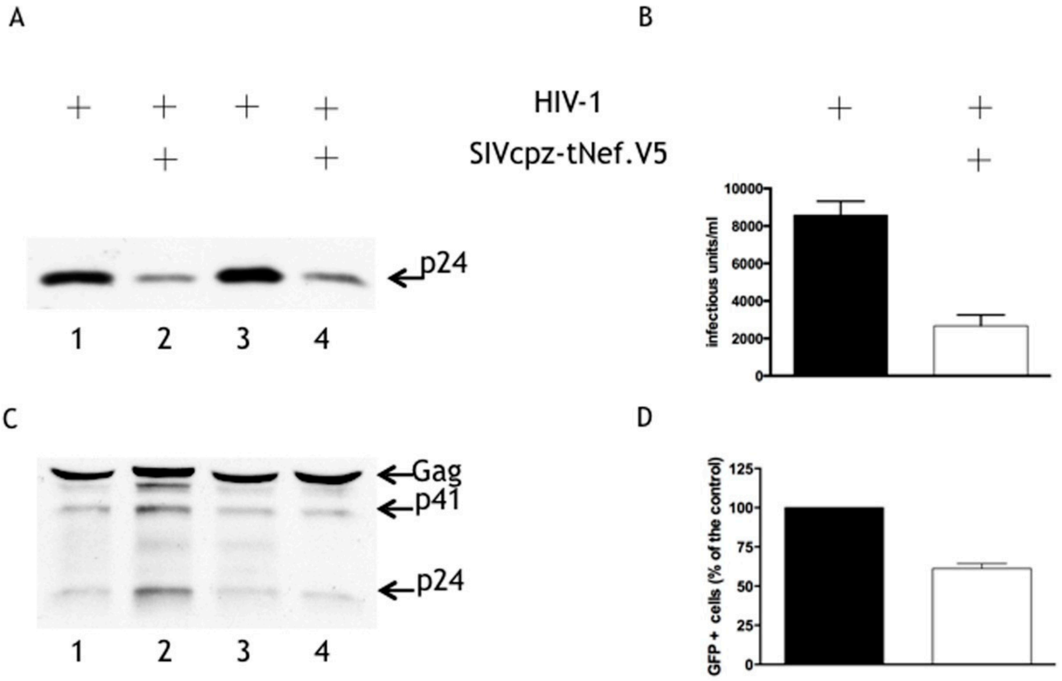

Figure S6. The SIVcpz truncated SIVcpz tNef peptide inhibits the release of HIV-1 progeny from Molt cells. Molt cells were co-transfected with the HIV-1WTgfp proviral plus the empty vector (lanes 1 and 3), and the SIVcpz tNef.V5 vector (lanes 2 and 4) at a 1:3 ratio. (**A**) Release of mature viral particles was evaluated by Western-blotting of concentrated viral particles from cell-free supernatants with polyclonal α–p24; (**B**) Viral stocks were normalized and viral infectivity was assayed by TZM-bl assay; (**C**) The expression of the Gag precursor and the processed p24 in cell lysates harvested 24 h after transfection was evaluated with polyclonal α-p24; (**D**) The percentage of GFP positive cells was verified by FACS. The values are the average of triplicate assays; error bars are the standard deviation of the mean.

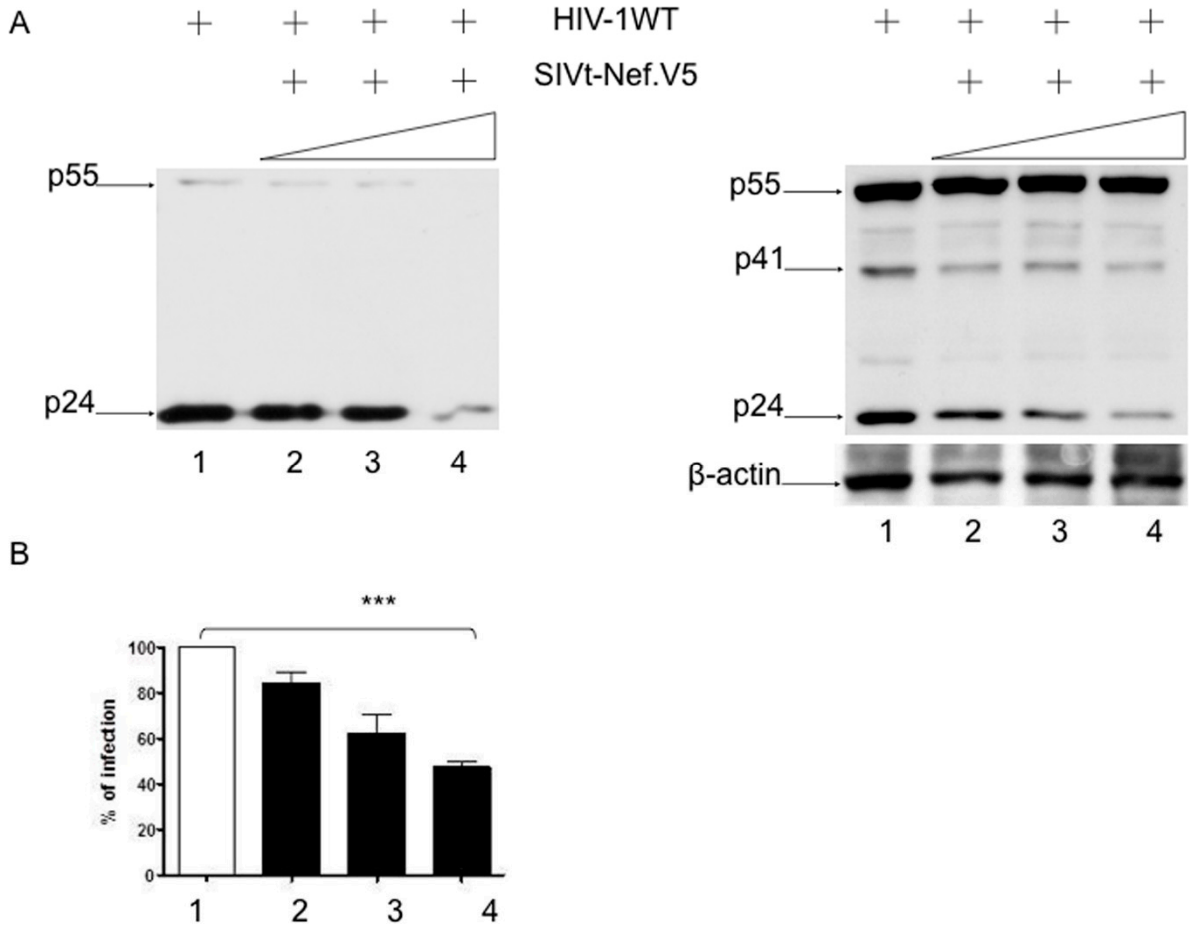

Figure S7. SIVcpz-tNef.V5 peptide inhibits HIV-1 infectivity. Hek-293T cells were co-transfected with 1 µg of HIV-1WT vector and 0, 1, 2 and 5 µg of the tNef vector (1:0, 1:1, 1:2 and 1:5 ratios). Differences in the amount of plasmid DNA in each transfection were compensated by the addition of empty vector (pcDNA3.1). Supernatants and lysate were collected 24 h post transfection. (**A**) Western Blot of viral and cell lysates using an α-CA antibody. Lanes 1 tthrough 4 are representative of 1:0, 1:1, 1:2 and 1:5 ratios, respectively; (**B**) Supernatants were used in infectivity assays with the TZM-bl indicator cells and the number of infected cells was counted and represented as percentage of the HIV-1WT control (*** $p = 0.043$ for the 1:2 ratio of HIV-1 and SIVcpz-tNef.V5 peptide; $p = 0.002$ for the 1:2 ratio of HIV-1 and SIVcpz-tNef.V5 peptide. Results represent three independent experiments and p-values < 0.05 were considered statistically significant.

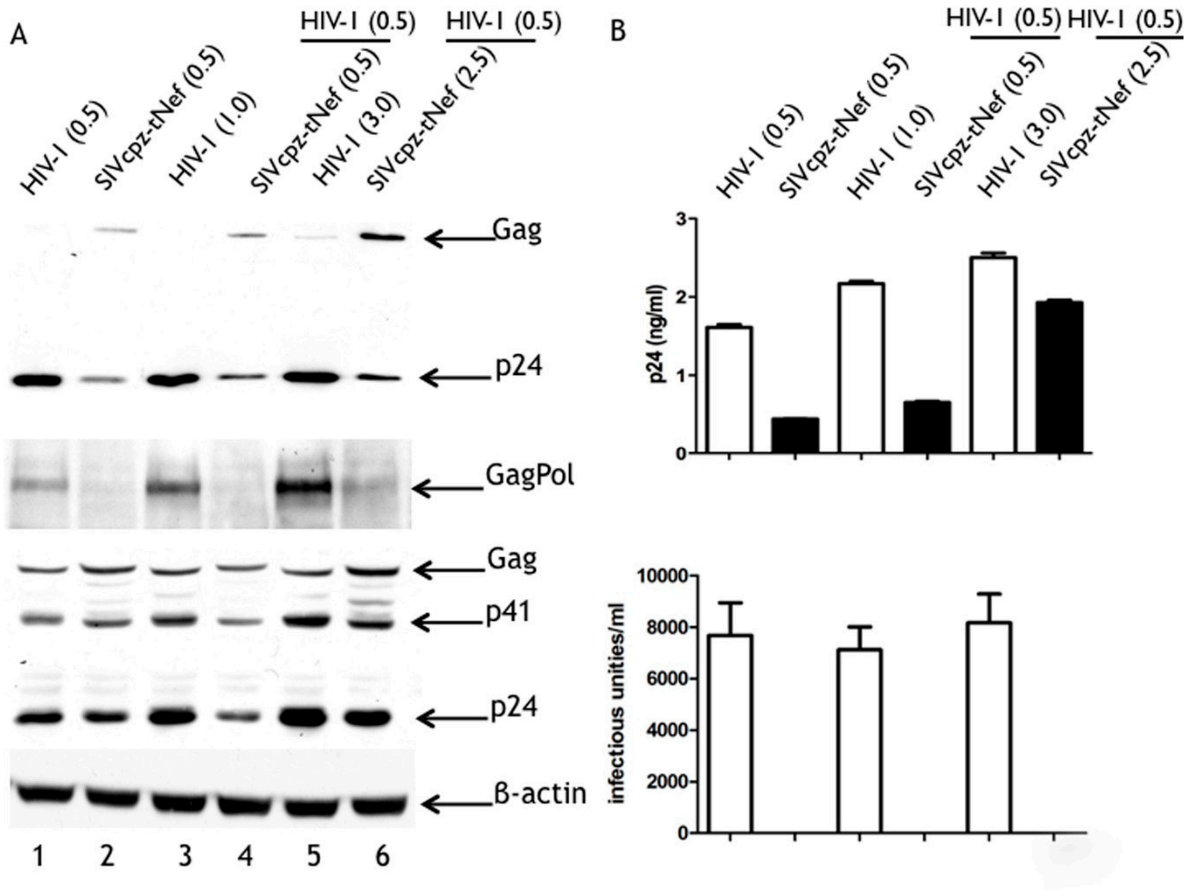

Figure S8. SIVcpz-tNef is a dominant negative for HIV-1WT. Hek-293T cells were co-transfected with different ratios of HIV-1WT to SIVcpz-tNef (1:0, 0:1, 2:0, 1:1, 6:0 and 1:5). Differences in the amount of plasmid DNA in each transfection were compensated by the addition of an empty vector. Cells lysates and cell-free supernatants were harvested 48 h after transfection. (**A**) Western-Blot of concentrated viral particles and cellular lysates, using n α-CA antibody to visualize viral proteins GagPol, Gag, MACA and CA; β-actin as loading control (bottom panel). (**B**) Viral antigen was measured by p24-ELISA (graphic on the top); viral stocks were normalized and viral infectivity was assayed by TZM-bl assay (graphic on the bottom). The values are the average of duplicate assays of each viral stock; error bars are the standard deviation of the mean.

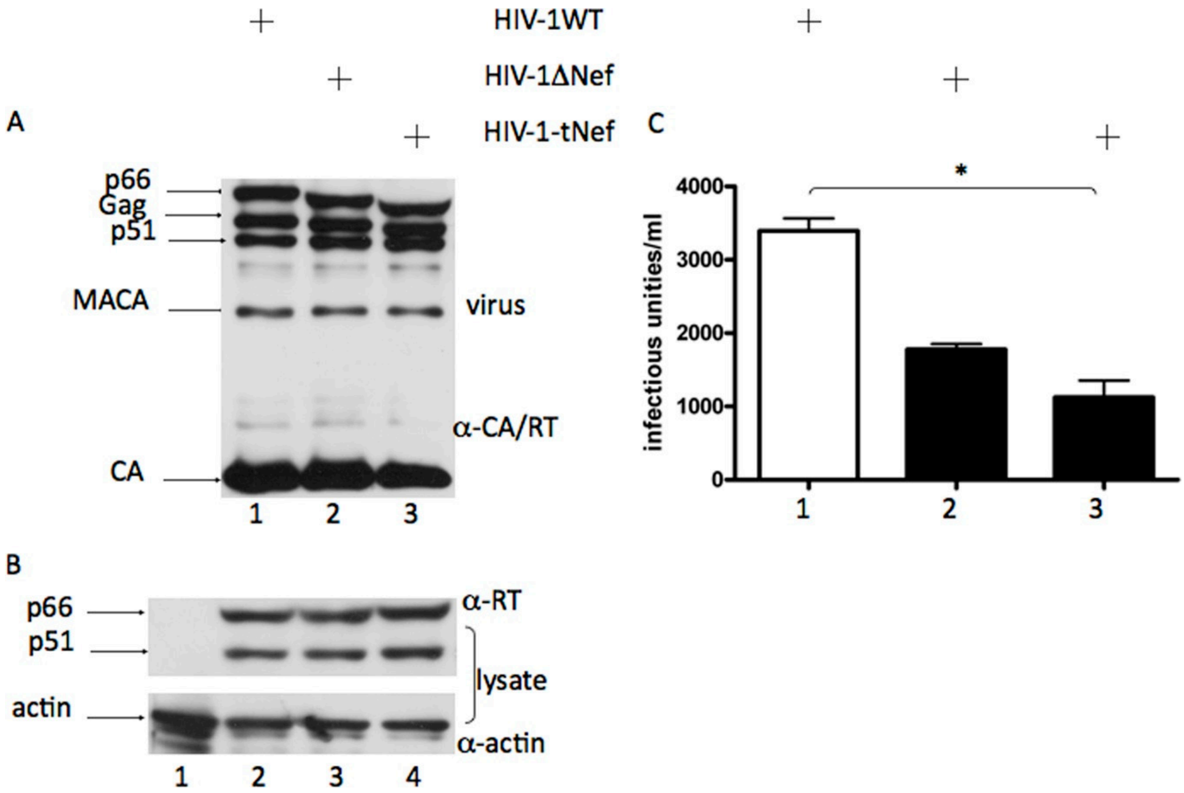

Figure S9. The truncated Nef peptide from HIV-1 (HIV-1-tNef) does not reproduce the effect of SIVcpz-tNef on processing and infectivity. HEK 293T cells were transfected with HIV-1WT, HIV-1ΔNef and HIV-1tNef proviral constructs. Supernatants and cell lysates were collected 24 hours post transfection. (**A**) Western Blot of viral particles concentrated by ultracentrifugation in a 20% sucrose cushion using a mixture of α-CA polyclonal antiserum which recognizes the Gag (55kDa), MACA (41kDa) precursors and the CA (24kDa) proteins, and α-RT antibody which recognizes the RT (51kDa and 66kDa) indicated by arrows; (**B**) Western Blot of cell lysates with α-RT and α-β-actin as loading control (**B**) Virus-containing supernatants were quantified by p24 ELISA and normalized viral stocks were used to inoculate TZM-bl indicator cell line for infectivity assay. The values are the average of triplicates of each viral stock; error bars are the standard deviation of the mean. * Asterisk indicates that the difference in infectivity of HIV-1ΔNef and HIV-1tNef to the HIV-1WT is statistically significant ($p = 0.002$ and $p = 0.045$, respectively).

© 2016 by the authors. Submitted for possible open access publication under the terms and conditions of the Creative Commons Attribution (CC-BY) license (http://creativecommons.org/licenses/by/4.0/).
